# A Sensor Localization and Orientation Method for OPM-MEG Based on Rigid Coil Structures and Magnetic Dipole Fitting Models

**DOI:** 10.3390/bioengineering12111198

**Published:** 2025-11-02

**Authors:** Weinan Xu, Wenli Wang, Fuzhi Cao, Nan An, Wen Li, Min Xiang, Xiaolin Ning, Ying Liu, Baosheng Wang

**Affiliations:** 1 School of Instrumentation and Optoelectronic Engineering, Beihang University, Beijing 100191, China; xwnan@buaa.edu.cn (W.X.); zb1917006@buaa.edu.cn (W.W.); caofuzhi@buaa.edu.cn (F.C.); liwen1997@buaa.edu.cn (W.L.); xiang_min@buaa.edu.cn (M.X.); ningxiaolin@buaa.edu.cn (X.N.); 2 National Institute of Extremely-Weak Magnetic Field Infrastructure, Hangzhou 310028, China; annan@buaa.edu.cn; 3 Hefei National Laboratory, Hefei 230088, China; 4 Hangzhou Lingci Medical Equipment Co., Ltd., Hangzhou 310057, China

**Keywords:** OPM-MEG, magnetic dipole fitting, sensor co-registration, rigid coil structure (RCS), weighted Frobenius norm, structural similarity index (SSIM)

## Abstract

High-precision sensor co-registration is a critical prerequisite for achieving high-resolution imaging in Optically Pumped Magnetometer–Magnetoencephalography (OPM-MEG) systems. The conventional magnetic dipole fitting method, essentially a multipole expansion approximation of a finite-size coil, exhibits accuracy that strongly depends on spatial geometric factors such as coil–sensor distance, dipole orientation, and the projection angle of the sensor’s sensitive axis. Moreover, the approximation error increases significantly when sensors are placed either too close to the coils or at an unfavorable angular coupling. To address this issue, we propose a sensor localization and orientation method that combines magnetic dipole-equivalent modeling with a rigid coil structure (RCS). The RCS provides stable geometric constraints and eliminates uncertainties introduced by scalp-attached coils. In addition, three objective functions (the standard Frobenius norm, a weighted Frobenius norm and the structural similarity index (SSIM)) are formulated to mitigate the imbalance caused by near-field strong signals and to improve stability under noise and error propagation. Simulation results demonstrate that both under ideal conditions and with assembly perturbations, the weighted Frobenius norm and SSIM methods consistently achieve position errors below 1 mm and orientation errors below 1°, which effectively suppress large outlier deviations and achieve better performance than the standard Frobenius norm. The results confirm the effectiveness of the proposed method in achieving both high accuracy and robustness. Beyond clarifying the primary factors influencing magnetic dipole approximation errors, this study provides a geometry-constrained and optimization-based framework, offering a feasible pathway toward the practical implementation of high-precision, multi-channel OPM-MEG systems.

## 1. Introduction

Magnetoencephalography (MEG) is a powerful technique for investigating neuronal activity in the human brain and has been increasingly applied in both basic neuroscience and clinical research [1,2,3,4,5,6,7,8,9,10]. Superconducting quantum interference devices (Low-Tc SQUIDs) have been the technological foundation of MEG systems for several decades and are well established now [11]. Nevertheless, their high operational costs and fixed sensor positions impose significant limitations in current MEG studies [12]. In recent years, wearable optically pumped magnetometers (OPMs) [13,14] and high-Tc SQUIDs [15,16] have emerged as promising alternatives for MEG [17,18,19]. Notably, the sensitivity of OPMs is not limited to neuroscience applications: recent advances have demonstrated that miniature, high-sensitivity OPMs can even achieve precise flow-rate measurements in magnetic particle detection [20].

Sensor co-registration, which aligns MEG sensors with the scalp in a unified coordinate system, is an essential step in localizing neuronal sources. Existing co-registration techniques primarily rely on optical methods and current-driven coil equivalents modeled as magnetic dipoles [21,22]. Optical tracking provides relatively high precision. However, it only determines the outer casing of the sensors rather than their true sensitive points. In contrast, coil-based methods directly address this limitation, making them indispensable for accurate source localization. Early implementations typically employed head position indicator (HPI) coils combined with an electromagnetic tracking system (e.g., Polhemus Fastrak), where HPI coils were placed at anatomical landmarks to establish a reference coordinate system [23,24].

Pfeiffer et al. [25,26] modeled HPI coils as magnetic dipoles attached directly to the scalp, systematically analyzing the effects of noise, magnetic moment amplitude, calibration errors, coil position errors, coil orientation errors and coil count on localization accuracy. Their simulations showed that magnetic moment and noise levels have a strong impact on the localization error which can be reduced by increasing measurement time. A ± 1% growth of calibration error induces up to 3 mm localization error. Positional error increases approximately linearly with coil–sensor distance and increasing the number of coils is able to reduce the positional error. Although their work discussed the influence of coil–sensor distance, it did not fully address other geometric parameters or the inherent approximation error of dipole modeling.

Despite these advances, the existing literature lacks a systematic analysis of how coil design parameters and coil–sensor spatial configurations affect dipole fitting accuracy. Most studies simply apply Biot–Savart law approximations without quantifying the deviation between real coil fields and dipole models. Consequently, uncharacterized approximation errors may introduce hardware-level biases at the outset of MEG experiments.

To solve this gap, we propose an integrated framework for OPM-MEG sensor co-registration that spans theoretical modeling, simulation and engineering implementation. First, we derive the sources of dipole approximation error from the Biot–Savart principle and define a quantitative metric for dipole fitting accuracy. We then establish a rigid coil structure (RCS) that imposes spatial geometric constraints, enabling a unified parameterization of sensor positions and orientations. Furthermore, we introduce three objective functions (standard Frobenius norm, weighted Frobenius norm and SSIM) and employ a hybrid local optimization strategy to improve fitting stability and accuracy. Simulation studies under both ideal geometry and perturbed conditions (0.5 mm positional and 0.5° angular deviations) are conducted to evaluate localization accuracy and robustness. Results show that the proposed approach achieves sub-millimeter and sub-degree accuracy under ideal conditions, while maintaining high robustness under assembly errors, significantly outperforming the conventional Frobenius norm method.

Overall, this study introduces both the modeling framework and optimization strategies for OPM-MEG co-registration, offering theoretical and technical support for the engineering deployment of high-precision, multi-channel MEG systems.

## 2. Principles and Methods

### 2.1. Magnetic Field Modeling in Space

The transformation between the coil array and sensor coordinate systems can be determined by analyzing their relative spatial relationship. This process is realized by driving currents through coils and measuring the corresponding magnetic fields at the sensors. The formulation is as follows. (The functions of all main parameters refer to Appendix A.)

For a closed loop carrying a steady current I, the magnetic field at any point in space (excluding points on the loop) along the sensor’s sensitive axis can be expressed as [27]:

(1) $$B_{c}=\frac{\mu_{0}In_{s}}{4\pi }\oint_{L}\frac{dr_{c}^{\prime }\times \left(r_{s}-r_{c}^{\prime }\right)}{{\left|r_{s}-r_{c}^{\prime }\right|}^{3}}$$

where $\mu_{0}$ is permeability of vacuum, L is the current loop path of the coil, $n_{s}$ represents the unit vector of the sensor’s sensitive axis, $r_{s}$ is the position vector of the sensor relative to the origin, and $r_{c}^{\prime }$ denotes a point on the current loop. When the sensor is located far from the current loop, the loop can be approximated as a point-like magnetic dipole characterized only by its magnetic moment. In this case, the magnetic field simplifies to:

(2) $$B_{d}=\frac{\mu_{0}}{4\pi }\left[\frac{3r_{c,s}(m_{c}\cdot r_{c,s})}{r_{c,s}^{5}}-\frac{m_{c}}{r_{c,s}^{3}}\right]n_{s}$$

where $r_{c,s}=r_{c}-r_{s}$ is the position vector of the sensor with respect to the coil, also referred to herein as the sensor observation direction, and $m_{c}$ is the magnetic moment vector of coil. The position of coil $r_{c}$ is $r_{c}=\left[x_{c},y_{c},z_{c}\right]$, and its orientation is ${\hat{r}}_{c}=(\theta_{c},\phi_{c})$. Assuming the sensor position is $r_{s}=\left[x_{s},y_{s},z_{s}\right]$ and the sensor orientation is ${\hat{r}}_{s}=(\theta_{s},\phi_{s})$, ${\hat{r}}_{c}$ and ${\hat{r}}_{s}$ are the unit vectors in the directions of $r_{c}$ and $r_{s}$, respectively. To simplify calculations and reduce the number of parameters, spherical coordinates are adopted for representing directional vectors. Expanding the vector dot products in Equation (2), we obtain:

(3) $$B_{d}=\frac{\mu_{0}\left|m_{c}\right|}{4\pi {\left|r_{c,s}\right|}^{3}}\left(3\cos\theta_{m}^{c,s}\cos\theta_{n}^{c,s}-\cos\theta_{c}^{s}\right)$$

where $\theta_{m}^{c,s}=∠\left(m_{c}\cdot r_{c,s}\right)$ is the angle between the coil’s magnetic moment direction and the observation direction, $\theta_{n}^{c,s}=∠\left(n_{s}\cdot r_{c,s}\right)$ is the angle between the sensor’s sensitive axis and the observation direction, and $\theta_{c}^{s}=∠\left(m_{c}\cdot n_{s}\right)$ is the angle between the magnetic moment and the sensor’s sensitive axis. Considering the impact of angular coupling on relative error, the relationship of the magnetic moment can be omitted in the analysis, thus we obtain:

(4) $$B_{d}\propto \frac{1}{{\left|r_{c,s}\right|}^{3}}\left(3\cos\theta_{m}^{c,s}\cos\theta_{n}^{c,s}-\cos\theta_{c}^{s}\right)$$

The error between the Biot–Savart law (Equation (1)) and dipole approximation (Equation (2)) can be written as:

(5) $$\varepsilon =\left|B_{c}-B_{d}\right|=\left|\frac{\mu_{0}\left|m_{c}\right|}{4\pi {\left|r_{c,s}\right|}^{3}}\left(3\cos\theta_{m}^{c,s}\cos\theta_{n}^{c,s}-\cos\theta_{c}^{s}\right)-B_{c}\right|$$

Since the dipole model is a first-order truncation of the multipole expansion, the dominant error term arises from the quadrupole component [28,29]:

(6) $$\varepsilon \left(r_{c,s}\right)∼ο\left(\frac{1}{r_{c,s}^{4}}\right)$$

To estimate the validity range of the dipole approximation, we evaluated the ratio of the quadrupole field magnitude to the primary dipole field under a typical coil–sensor configuration (radius of the coil $r_{c}$ = 10 mm, $r_{c,s}$ = 30–50 mm). The result indicates that the quadrupole term contributes less than 1.5% of the dipole field magnitude when $r_{c,s}/r_{c}$ ≥ 3, which is well within the spatial region of interest for OPM-MEG measurements. Therefore, the dipole approximation used in this study is sufficient for accurately modeling the magnetic field generated by the rigid coil system.

From this theoretical analysis, four key parameters influencing the approximation error can be identified:

**Coil–sensor distance** $\left|r_{c,s}\right|$**:** The dipole field decays as ${\left|r_{c,s}\right|}^{3}$ while the error term is dominated by quadrupole term. As the sensor approaches the coil, the dipole approximation degrades rapidly.
**Angle** $\theta_{m}^{c,s}$**:** Governs the radial component of the dipole field. When $\theta_{m}^{c,s}=90^{\circ }$, the magnetic moment is perpendicular to the observation direction. In this case, the radial component of the dipole field (the first term in Equation (2)) becomes zero, while the tangential component (the second term) remains non-zero. Therefore, the total dipole field does not vanish but changes direction.
**Angle** $\theta_{n}^{c,s}$**:** Determines the projection of the dipole field onto the sensor’s sensitive axis. When $\theta_{n}^{c,s}=90^{\circ }$, the sensor’s sensitive axis is perpendicular to the observation direction, and the projection of the dipole field onto the sensor’s sensitive axis becomes minimal, although the total field itself is not zero.
**Angle** $\theta_{c}^{s}$**:** Describes the coupling between the dipole moment and the sensor axis. When $\theta_{c}^{s}=90^{\circ }$ or $\cos\theta_{c}^{s}=0$, the coupling is maximized, while intermediate values can produce destructive interference, amplifying local modeling errors.

This theoretical derivation quantifies the magnetic dipole approximation error and identifies the four primary influencing factors: the coil–sensor distance $\left|r_{c,s}\right|$, and the three angles $\theta_{m}^{c,s}$, $\theta_{n}^{c,s}$, and $\theta_{c}^{s}$. The analysis confirms that the error is not a simple function of distance alone but is governed by a complex, nonlinear coupling of these geometric parameters. This intricate interdependence makes the error highly sensitive to the specific spatial configuration and difficult to predict or model accurately for arbitrary coil–sensor pairs. Consequently, in practical systems where coil positions are not rigidly constrained (scalp-attached coils), the unpredictable variation in these parameters introduces significant and unstable errors into the dipole fitting process, ultimately degrading the accuracy of sensor co-registration. This fundamental challenge motivates the engineering solution proposed in the following section.

**Figure 1:**
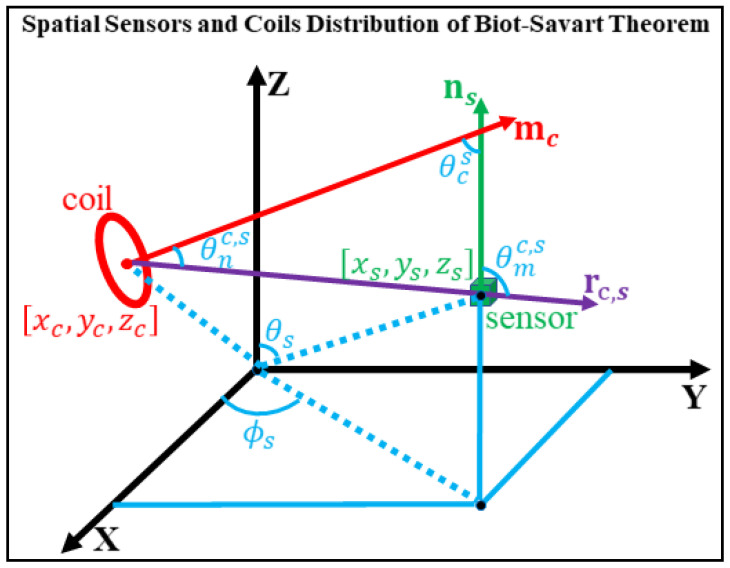
Spatial distribution of sensors and coils based on the Biot–Savart law.

The overall structure of the rigid coil system and sensor arrangement is illustrated in Figure 1.

### 2.2. Selection of Objective Functions

In the process of determining sensor position and orientation through multi-parameter nonlinear least-squares fitting, it is essential to select an appropriate objective function. Because the raw signals contain substantial spatial noise, band-pass filtering must be applied during preprocessing. However, such filtering alters the overall amplitude of the original signals. Thus, a scaling factor must be introduced into the computation. In this framework, the task is to localize the sensor relative to the coils. While the positions and orientations of the coils are known quantities. In addition, the coil and sensor numbers must also be taken into consideration.

There are total of six unknown parameters ($\theta_{s}$, $\phi_{s}$, $x_{s}$, $y_{s}$, $z_{s}$, $k\left[I(t)\right]$) that need to be calculated. Their theoretical measured values can be obtained from Equation (2).

(7) $$B_{c,s}^{*}=\frac{\mu_{0}}{4\pi }(\frac{3r_{c,s}^{*}(m_{c}^{*}\cdot r_{c,s}^{*})}{{r_{c,s}^{*}}^{5}}-\frac{m_{c}^{*}}{{r_{c,s}^{*}}^{3}})n_{s}$$

where $B_{c,s}^{*}=$$B_{c,s}^{*}(\theta_{s},\phi_{s},x_{s},y_{s},z_{s},k\left[I(t)\right])$, $r_{c,s}^{*}=r_{c,s}^{*}(x_{s},y_{s},z_{s})$, I(t) denotes the time-dependent current in the coil, $k\left[I(t)\right]$ represents a scaling factor proportional to the coil driving current I(t). The solution is derived via a nonlinear minimization algorithm applied to a temporal segment of the measured magnetic field $B_{c,s}^{*}$.

(8) $$f(\theta_{s},\phi_{s},x_{s},y_{s},z_{s},k)=\arg\min f(B_{c,s}^{*},B_{c,s})$$

To address varying signal amplitudes and noise distributions, three categories of objective functions are introduced:

(9) $$f_{F}(\theta_{s},\phi_{s},x_{s},y_{s},z_{s},k)=\frac{{\left\|B_{c,s}^{*}-B_{c,s}\right\|}_{F}}{{\left\|B_{c,s}\right\|}_{F}}$$

The Frobenius norm fully utilizes all data points within the measurement window. However, it suffers from imbalance: large-amplitude signals (typically from near-field coils) dominate the optimization, suppressing contributions from weaker signals. To solve this imbalance, a weighting scheme designed to balance near-field and far-field signal contributions is applied. Although the distance itself is not explicitly included in the weighting function, the normalization implicitly compensates for signal amplitude variations caused by coil–sensor spacing:

(10) $$f_{W}(\theta_{s},\phi_{s},x_{s},y_{s},z_{s},k)=\frac{{\left\|{\bar{B}}_{c,s}^{*}-{\bar{B}}_{c,s}\right\|}_{F}}{{\left\|{\bar{B}}_{c,s}\right\|}_{F}}$$

(11) Bc,s∗=Bc1,s1∗Bc1,s2∗…Bc1,sj∗Bc2,s1∗Bc2,s2∗…Bc2,sj∗⋮⋮⋱⋮Bci,s1∗Bci,s2∗…Bci,sj∗Bc,s=Bc1,s1Bc1,s2…Bc1,sjBc2,s1Bc2,s2…Bc2,sj⋮⋮⋱⋮Bci,s1Bci,s2…Bci,sjB¯c,s∗=BsumBc1,s1∗BsumBc1,s2∗…BsumBc1,sj∗BsumBc2,s1∗BsumBc2,s2∗…BsumBc2,sj∗⋮⋮⋱⋮BsumBci,s1∗BsumBci,s2∗…BsumBci,sj∗B¯i,j=BsumBc1,s1BsumBc1,s2…BsumBc1,sjBsumBc2,s1BsumBc2,s2…BsumBc2,sj⋮⋮⋱⋮BsumBci,s1BsumBci,s2…BsumBci,sj

(12) $$B_{sum}=\sum_{i=1}^{m}\sum_{j=1}^{n}B_{c_{i},s_{j}}$$

Owing to the physical characteristics of magnetic dipoles, the accuracy of the dipole approximation increases with sensor-coil distance. To mitigate the larger approximation errors occurring in the near-field region, the objective function is modified by assigning greater weights to far-field signal channels and smaller weights to near-field channels. Specifically, both the measured and theoretical magnetic field matrices are normalized using a constant weighting factor, resulting in a weighted matrix. For consistency, this factor is defined as the sum of all elements in the measured magnetic field matrix $B_{c,s}$.

Instead of measuring absolute magnitude differences, SSIM evaluates the structural similarity between two datasets, which makes it less sensitive to the scaling effects introduced by filtering and noise [30].

(13) $$f_{\mathrm{ssim}}(\theta_{j}^{s},\phi_{j}^{s},x_{j}^{s},y_{j}^{s},z_{j}^{s},k_{j}^{s})=1-\mathrm{SSIM}(B_{c,s},B_{c,s}^{*})$$

The SSIM metric follows the standard formulation introduced in [30]. SSIM parameter settings refer to Appendix B.

### 2.3. Ideal Single-Turn Coil Model

For simplicity, each coil is modeled as a single-turn circular loop with negligible wire thickness. Its parametric representation in Cartesian coordinates is:

(14) $$\left\{\begin{array}{l} x_{cl}=x_{cl0}+\left(r_{cl}\cos\alpha_{cl}\right){\hat{u}}_{x}+\left(r_{cl}\sin\alpha_{cl}\right){\hat{v}}_{x}\\ y_{cl}=y_{cl0}+\left(r_{cl}\cos\alpha_{cl}\right){\hat{u}}_{y}+\left(r_{cl}\sin\alpha_{cl}\right){\hat{v}}_{y}\\ z_{cl}=z_{cl0}+\left(r_{cl}\cos\alpha_{cl}\right){\hat{u}}_{z}+\left(r_{cl}\sin\alpha_{cl}\right){\hat{v}}_{z} \end{array}\right.,\alpha_{cl}\in [0,2\pi ]$$

The position vector of a single sensor in space is denoted as $r_{s}$ = $\left[x_{s},y_{s},z_{s}\right]$. By substituting sensor $r_{s}$ and coil $r_{c}$ into Equation (1) and Equation (2), respectively, the magnetic field values generated at any arbitrary position in space are calculated and denoted as $B_{cl}$ and $B_{dl}$. The computational process is illustrated below:

(15) $$B_{cl}=\frac{\mu_{0}I{\hat{n}}_{s}}{4\pi }\oint_{L(\alpha_{cl})}\frac{dr_{cl}\times \left(r_{s}-r_{cl}\right)}{{\left|r_{s}-r_{cl}\right|}^{3}}$$

where $dr_{cl}=r_{cl}(-\hat{u}\sin\alpha_{cl}+\hat{v}\cos\alpha_{cl})d\alpha_{cl}$.

Equation (15) provides the spatial magnetic field generated by a single-turn coil according to the Biot–Savart law, which serves as the ideal theoretical reference. The corresponding field of the magnetic dipole approximation is subsequently derived, with its value, denoted as $B_{dl}$, computed from Equation (2) as follows:

(16) $$B_{dl}=\frac{\mu_{0}}{4\pi }(\frac{3r_{c,s}(m_{l}\cdot r_{c,s})}{{\left|r_{c,s}\right|}^{5}}-\frac{m_{l}}{{\left|r_{c,s}\right|}^{3}}){\hat{n}}_{s}$$

where $r_{c,s}=r_{s}-r_{cl}$ and the magnetic moment of the single-turn coil is $m_{l}=\pi Ir_{cl}^{2}{\hat{m}}_{l}$, ${\hat{m}}_{l}$ is the unit vector of $m_{l}$.

As the magnetic dipole model is only an approximation, discrepancies are unavoidable between its generated field and the ideal theoretical field. To quantitatively evaluate this error, a parameter $E=\left|B_{c}/B_{d}-1\right|$ is introduced to characterize the goodness of fit between the equivalent magnetic field of the dipole model for a single-turn coil and the corresponding ideal field. The value of E approaching zero indicates a closer agreement with the ideal model.

## 3. Simulation Analysis and System Design

### 3.1. Simulation of Magnetic Dipole Fitting in RCS Channels

To improve the fitting accuracy of magnetic dipole equivalent modeling, we developed a rigid coil structure (RCS). In addition to serving as a mechanical framework for mounting multiple coils, the RCS fundamentally addresses the problem of accuracy degradation under multi-parameter coupling that limits conventional approaches. Errors in dipole approximation are affected nonlinearly by coil–sensor distance, relative orientation, and projection angle. Analyzing these parameters independently is computationally expensive and risks neglecting cross-effects. The primary advantage of the RCS is that it fixes both coil and sensor spatial information within the same coordinate system. By predefining all geometric parameters, the RCS reduces modeling uncertainties in practical measurements and eliminates multiple error sources caused by adhesive placement on the scalp. Consequently, incorporating structural priors removes variable interference, ensuring that dipole fitting under known geometries is stable and reliable, thereby providing a solid foundation for subsequent localization and error analysis.

The RCS is fabricated from photosensitive resin via 3D printing and comprises two main parts: a base and a coil-array frame. The base conforms to the forehead contour and is compatible with the previously designed 85-channel rigid helmet, with ten reference markers embedded. The coil-array frame consists of eight arc-shaped supports, each fitted with 3~5 evenly spaced threaded studs. These studs allow coils of different specifications to be mounted using nuts, ensuring that each coil is positioned at least 5 cm from the nearest sensor.

**Figure 2:**
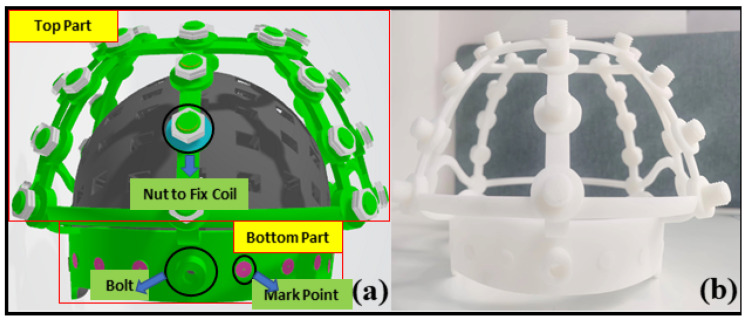
Custom-designed RCS assembly. (**a**) Schematic of the RCS design; (**b**) Fabricated RCS prototype.

The RCS accommodates 35 coils with fixed positions and magnetic moment orientations. The rigid helmet serves primarily as a reference framework: when rigidly attached to the RCS, both coils and sensors share a common coordinate system, with all spatial information predefined. This simulation aims to assess the effect of dipole fitting accuracy on sensor localization. Figure 2 illustrates the 3D model of the RCS and its fabricated prototype.

The rigid coil structure (RCS) was fabricated using a 3D-printed non-magnetic resin (relative permeability μᵣ ≈ 1.0, dielectric constant εᵣ ≈ 2.8) to minimize magnetic interference and dielectric coupling. Copper wire was embedded within the printed substrate under low-temperature curing (<60 °C) to reduce thermal deformation and maintain geometric stability. The mechanical interface between the helmet and the coil frame was designed with a tolerance of ±0.2 mm, ensuring consistent positioning and alignment across repeated measurements.

The material and geometric characteristics of the RCS may influence local magnetic field uniformity, particularly near the coil–sensor interface. Although the non-magnetic resin contributes negligible distortion to the modeled magnetic field, future studies will include more detailed simulations and experimental tests to quantitatively evaluate the influence of material and structural parameters on magnetic field distribution and system robustness.

The rigid helmet accommodates 85 sensor slots, while the RCS supports up to 35 coils. Using the equivalent dipole approach, a total of 85 × 35 = 2975 coil–sensor combinations are simulated. Each sensor is represented by its sensitive-point coordinates and sensitive-axis unit vector (85 sets), and each coil by its center coordinates and dipole unit vector (35 sets). All vectors are expressed in Cartesian coordinates.

The simulated coil is a single-turn ring with an inner diameter $r_{coil}$ of 5 mm, neglecting the wire diameter. A current of 1 mA is applied, and the direction of the magnetic moment is specified. The resulting magnetic field which calculated under these conditions is denoted as $B_{c}$. The corresponding fitting accuracy for each dataset is designated as $E=\left|B_{c}/B_{d}-1\right|$.

**Figure 3:**
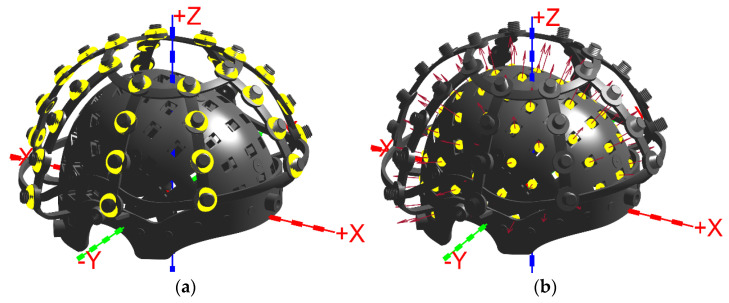
Combined RCS and rigid helmet showing the coordinate system, sensors, and coils. (**a**) Coil depicted as a yellow ring; (**b**) Sensor depicted as a yellow point, with the red arrow indicating the orientation of the sensitive axis.

The simulated coil and sensor configuration used for evaluating the RCS-based localization framework is illustrated in Figure 3.

**Figure 4:**
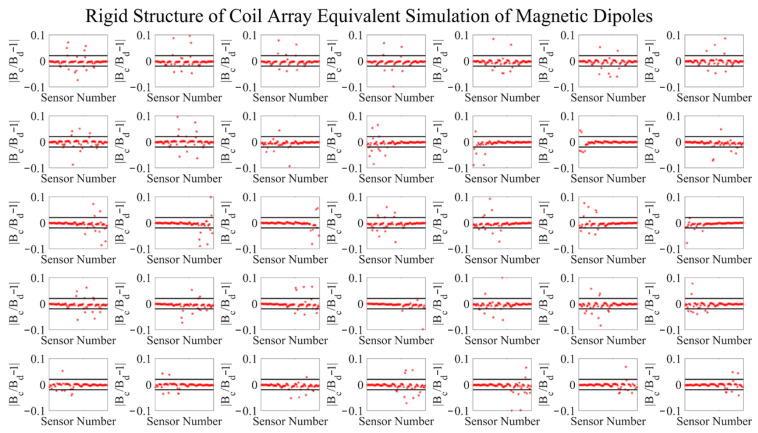
Equivalent dipole simulation results of the RCS.

Figure 4 presents the dipole fitting accuracy for all 35 coils, with each point representing a sensor-coil combination. For a clearer statistical summary, the fitting accuracies of all 2975 points are listed in Table 1.

Table 1 indicates that for more than 80% of the 2975 coil–sensor pairs, the equivalent dipole error is below 0.01, with over 94% below 0.05, confirming the practical utility of the RCS design. Although most pairs achieve high accuracy, a few exhibits relatively low fitting accuracy due to angular constraints. These errors can be mitigated by increasing the number of coils or by applying the fitting objective functions described above.

To further assess the stability and accuracy advantages of the RCS in magnetic dipole modeling, an additional simulation was conducted following the RCS configuration tests. Specifically, a set of coils mimicking direct attachment to the human scalp was constructed to simulate practical scenarios, and the results were compared with those obtained using the RCS under identical conditions

Since this study has not yet incorporated a detailed human MRI model or realistic head phantom, the simulation was based on the spatial distribution of the 35 coils in the original RCS. The coil array was first fitted to an approximate spherical surface, with the sphere center computed as the geometric reference point. Using this center as a reference, all coils were then uniformly scaled inward while preserving their magnetic moment orientations, shifting their positions closer to the inner surface of the rigid helmet. This transformation maintained the relative angular distribution among the coils and produced a reasonable and comparable scalp-attached model.

For coil modeling, each coil was represented as a single-turn circular loop with a radius of 5 mm, neglecting wire thickness and with a known dipole orientation. Magnetic fields were computed using line-integral methods. The sensor configuration remained unchanged, consisting of the original 85-channel array with known spatial coordinates and sensitive axes.

Within the simulation framework, magnetic fields were computed at the 85 sensor positions using the scalp-attached coil configuration. These fields were then fitted using the magnetic dipole model, and relative error metrics were calculated. In total, 85 × 35 = 2975 data pairs were generated, which were subsequently partitioned and statistically compared with the results obtained under the RCS configuration.

**Figure 5:**
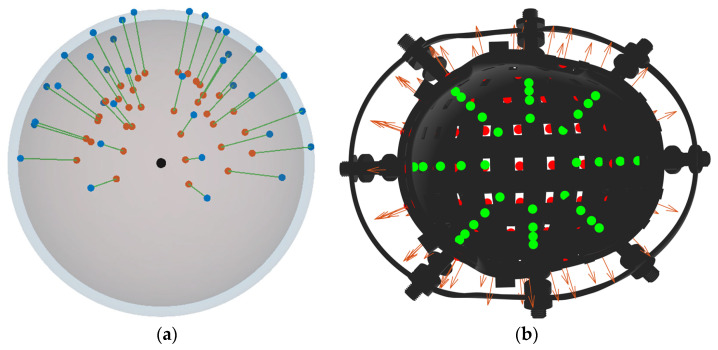
Coil placement near the inner surface of the helmet (scalp-attached configuration). (**a**) Blue points indicate the initial coil positions before contraction; red points indicate positions after contraction; black points indicate the center of the fitted sphere. (**b**) Green points indicate the schematic coil positions relative to the inner helmet surface after contraction.

Figure 5 shows the comparative localization errors under different noise conditions.

Table 2 demonstrate that the RCS achieves substantially higher fitting accuracy than the scalp-attached configuration. Specifically, within the high-accuracy range (<0.01), the RCS attains 80.24% coverage, compared to 52.24% for the scalp-attached coils. These findings confirm the advantage of the rigid structure in reducing geometric uncertainties and enhancing the stability of dipole modeling.

### 3.2. Simulation Analysis of Sensor Localization and Orientation Using RCS

This section examines the impact of dipole fitting accuracy on the spatial localization and orientation of sensors. In the simulations, magnetic fields calculated using the Biot–Savart law (Equation (1)) serve as the ground truth, while fields obtained from the dipole approximation (Equation (2)) are treated as measured values. Sensor position and orientation errors are subsequently computed.

To account for the variation in dipole fitting accuracy, three experimental groups were conducted. In each group, 20 coils were used to localize 10 sensors, yielding 200 data points per group. The coil–sensor pairs were selected to cover all accuracy intervals (equivalent fitting error <0.01, <0.02, <0.03, <0.04, <0.05, and >0.05), although over 80% of the data naturally fell within the <0.01 range. The coil inner radius was set to 5 mm, following the typical design parameters reported by [26] and the effective current was 1 mA, since the absolute current amplitude does not affect the relative accuracy of magnetic dipole fitting due to the field’s linear proportionality to current.

The overall workflow was as follows: magnetic fields at the 10 sensors were first computed using the Biot–Savart law as the reference dataset. Localization was then performed using the dipole formulation with coil positions provided as known inputs. Three objective functions-standard Frobenius norm, weighted Frobenius norm and SSIM were employed to estimate sensor positions and orientations via least-squares optimization.

**Figure 6:**
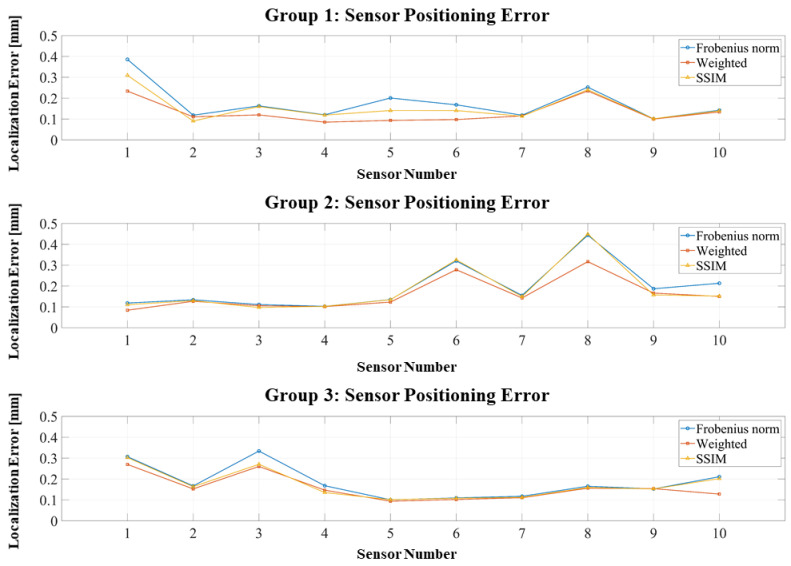
Positioning errors of three localization experiments using the RCS-based dipole approximation.

As shown in Figure 6, position errors across all three groups remain below 0.4 mm, confirming that the RCS design provides localization accuracy sufficient for co-registration purposes. Regarding the objective functions, both the weighted Frobenius norm and SSIM outperform the standard Frobenius norm, although the improvement from the weighted method is marginal, as the RCS inherently eliminates large-error channels.

**Figure 7:**
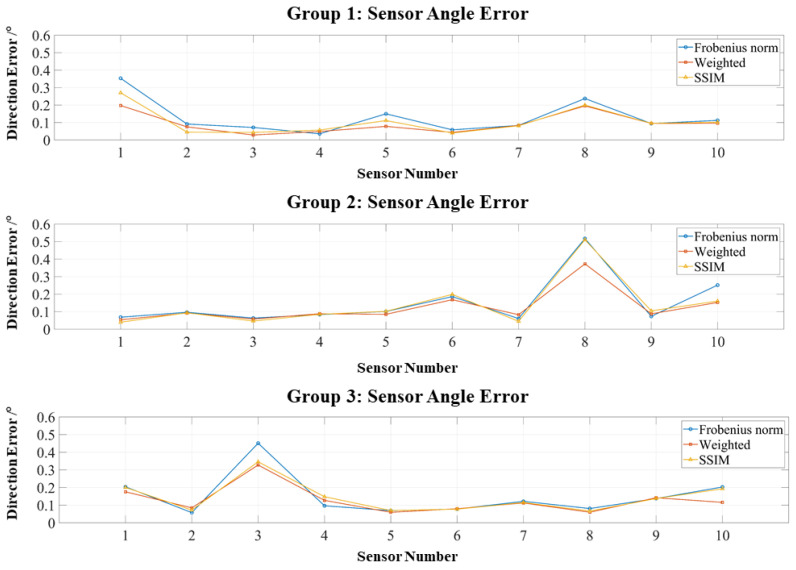
Orientation errors of three localization experiments using the RCS-based dipole approximation.

Figure 7 presents similar results for orientation errors: all remain below 1°, with most concentrated under 0.5°, further demonstrating the robustness of the RCS framework.

The results in Table 3 indicate that, under ideal geometric conditions, RCS-based dipole fitting attains high accuracy across all objective functions: average position errors remain below 0.2 mm, and orientation errors stay within 1°. The weighted Frobenius norm achieves the best performance, particularly in reducing angular errors, while SSIM provides stable results comparable to the weighted Frobenius norm. Furthermore, the error distribution is highly consistent across coil–sensor pairs, reflecting the stability and reproducibility of RCS-constrained dipole modeling.

### 3.3. Simulation with Assembly Errors

To further assess robustness under realistic conditions, an additional experiment introduced assembly errors to simulate inevitable coil misalignments during practical installation. Each coil’s ideal parameters were perturbed by random deviations of up to 0.5 mm in position and 0.5° in orientation. The deviations (0.5 mm in position and 0.5° in orientation) were set according to the manufacturing tolerances provided by the 3D-printing manufacturer of the RCS. The perturbed configuration was used to generate “true” magnetic fields, while the localization algorithm assumed the ideal coil geometry, thereby simulating real-world tolerance-induced mismatches.

**Figure 8:**
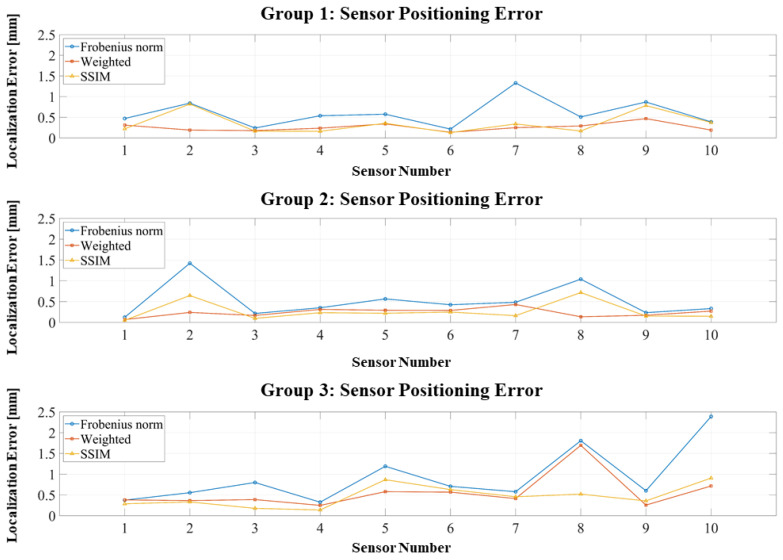
Positioning errors of three localization experiments with assembly perturbations.

**Figure 9:**
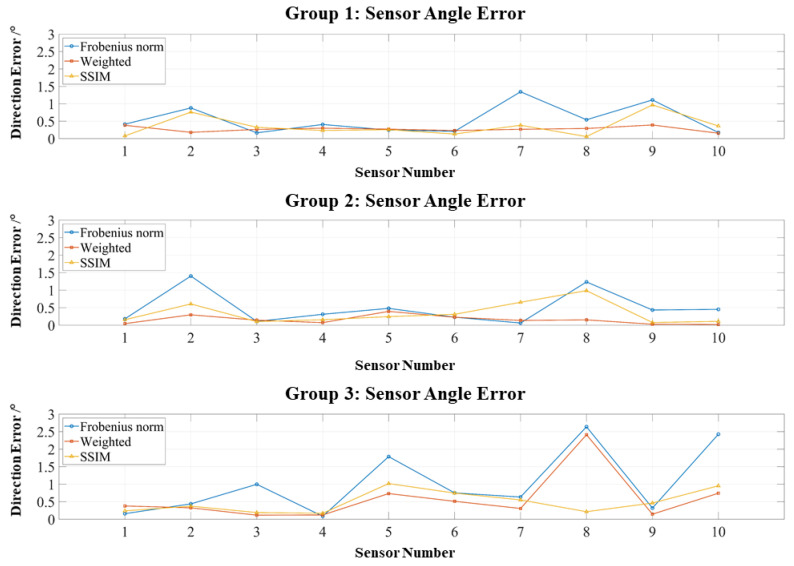
Orientation errors of three localization experiments with assembly perturbations.

As shown in Figure 8, the introduction of perturbations shifts the error distributions upward. However, most position errors remain within 1 mm. Both the weighted Frobenius norm and SSIM produce concentrated error distributions and suppress large outliers, in contrast to the more dispersed errors observed with the standard Frobenius norm. Figure 9 presents similar trends for orientation errors: The weighted Frobenius norm and SSIM generally maintain orientation errors below 0.6°, although occasional outliers exceed this threshold; such outliers are more frequent and larger for the standard Frobenius norm.

Table 4 confirms that, although all methods exhibit increased errors under perturbations, the weighted Frobenius norm and SSIM maintain strong robustness: position errors remain mostly below 0.6 mm, and orientation errors generally stay under 0.6°. In contrast, the standard Frobenius norm experiences substantial degradation, with errors approaching 1 mm and 1°. These results underscore the combined advantage of RCS geometric constraints and optimized objective functions in mitigating error propagation under realistic assembly tolerances.

Across both ideal and perturbed conditions, the proposed RCS-based dipole equivalent method consistently achieves high accuracy and stability in sensor localization and orientation. Under ideal conditions, position errors remain below 0.2 mm and orientation errors below 1°. Even with ±0.5 mm positional and ±0.5° angular perturbations, the weighted Frobenius norm and SSIM maintain errors under 0.6 mm and 0.6°, effectively suppressing large-error outliers. These results demonstrate that the RCS framework provides superior geometric stability and error control, enabling OPM-MEG systems to attain millimeter-level accuracy and sub-degree orientation precision under practical assembly constraints.

## 4. Discussion

This study systematically proposes and validates a high-accuracy, high-robustness sensor co-registration method for OPM-MEG systems, based on a rigid coil structure (RCS) combined with magnetic dipole equivalent modeling. Simulation results confirm that the method achieves excellent performance under both ideal and assembly error conditions, providing essential technical support for the engineering deployment of OPM-MEG systems. The main findings, significance, and limitations are discussed below.

First, the core innovation of this work lies in introducing strong geometric priors through the RCS. Compared with the scalp-attached coil method proposed by Pfeiffer et al. [25,26], in which coils are directly fixed to the scalp surface, the RCS integrates the coil array and sensor holder into a single rigid assembly, with all coil positions and orientations precisely predefined. Simulation comparisons (Table 1 and Table 2) clearly show that this rigid design reduces average dipole fitting error by nearly 30 percentage points (80.24% within <0.01 for RCS versus 52.24% for scalp-attached coils). This fundamentally eliminates geometric uncertainties inherent in adhesive coil placement, providing a stable and reliable physical basis for subsequent localization algorithms. Solving this co-registration accuracy problem is a critical prerequisite for fully leveraging the high sensitivity of OPM sensors in complex spatial configurations.

Second, in terms of optimization strategies, this study demonstrates the superiority of the weighted Frobenius norm and the structural similarity index (SSIM) over the standard Frobenius norm. Under ideal conditions, all three achieve millimeter-level precision (Table 3). However, when assembly errors are introduced, the Frobenius norm shows severe performance degradation (with average position error up to 0.93 mm), whereas weighted Frobenius and SSIM maintain stable performance, keeping errors below 0.6 mm (Table 4). This indicates that the Frobenius norm is highly sensitive to dominant near-field channels. In contrast, the weighted Frobenius norm balances channel contributions by assigning greater weight to far-field signals, while SSIM emphasizes structural consistency rather than pointwise amplitude, making it less sensitive to noise and scaling effects. Both methods effectively mitigate model mismatch and noise, enhancing the robustness of the localization process.

To better situate this work within the context of existing OPM-MEG calibration techniques, it is necessary to compare the proposed RCS-based method with two representative studies by Iivanainen et al. (2022) and Hill et al. (2025) [31,32]. Iivanainen et al. proposed a calibration approach using large external electromagnetic coils whose magnetic fields were modeled with a vector spherical harmonics (VSH) expansion. Their method enabled simultaneous estimation of sensor position, orientation, and gain, achieving approximately 3.3 mm average localization error for OPM arrays. Hill et al. later introduced two practical systems for wearable MEG calibration—the HALO head-mounted coil array and a matrix coil embedded in the shielded room—achieving localization accuracy of around 2 mm while also correcting sensor gain. In contrast, the Rigid Coil Structure (RCS) proposed in this study integrates small calibration coils directly into a rigid helmet to maintain fixed coil–sensor geometry. This design minimizes uncertainties caused by coil displacement and provides a stable mechanical reference for repeated localization. Furthermore, the RCS method explicitly analyzes the error between the magnetic dipole approximation and the full Biot–Savart field and employs two dedicated optimization functions—a distance-weighted Frobenius norm and a Structural Similarity Index (SSIM)—to enhance robustness against assembly perturbations. Although the present results are based on simulations, the RCS approach demonstrates sub-millimeter accuracy, indicating higher intrinsic geometric precision compared with existing coil-based methods and offering a complementary framework that can be extended to include gain calibration in future work.

Furthermore, the quantified relationship between dipole approximation error and spatial geometric parameters (distance, angular coupling, and projection effects) provides key theoretical guidance for future coil array design and sensor layout in OPM-MEG systems. These insights enable pre-optimization of spatial configurations to minimize fitting error at the design stage, thereby improving localization accuracy at the source. This theoretical contribution is particularly valuable for scaling up OPM arrays to large, high-density systems.

The performance of the proposed objective functions must be considered in the context of sensor noise. Theoretically, sensor noise could diminish the relative advantage of the weighted Frobenius norm over the standard norm. The standard norm exhibits inherent robustness to noise in far-field channels, as its optimization is dominated by the high-amplitude—and consequently high signal-to-noise ratio (SNR)—signals from near-field coil–sensor pairs.

However, the rigid coil structure (RCS) design ensures that all operational coil–sensor distances are maintained within a pre-optimized range, guaranteeing a sufficiently high SNR in practice. Consequently, the primary challenge addressed here is not insufficient SNR, but the dominant, systematic bias introduced by the breakdown of the magnetic dipole approximation in the near-field. This model error, if unmitigated, compromises localization accuracy more significantly than stochastic sensor noise.

Therefore, while sensor noise is present, its impact is secondary to the substantial model-based errors from unfavorable geometries. The weighted Frobenius norm and SSIM were specifically designed to counteract this dominant error source. The superior accuracy and robustness of these methods are thus expected to be preserved under the typical experimental SNRs ensured by the RCS framework.

### Limitations and Future Work

Although the proposed co-registration framework demonstrates high accuracy and robustness under both ideal and assembly error conditions, we acknowledge that the computational experiments presented in this study are limited in scope. The number of simulated configurations and the range of parameter constraints were intentionally restricted to isolate the core effects of the rigid coil structure and the magnetic dipole–based fitting method. As a result, the current results primarily serve as a proof-of-concept validation rather than a comprehensive evaluation of all possible conditions.

A major limitation of this study is that all validation results remain at the simulation level. Due to ongoing hardware preparation and the limited availability of OPM-MEG facilities, realistic head or MRI-based phantom data were not yet incorporated into the analysis. Consequently, the current results primarily serve as a theoretical and computational verification of the proposed framework.

Nevertheless, experimental validation is indispensable for assessing the real-world robustness and geometric accuracy of the co-registration algorithm. To address this limitation, a physical validation platform is currently under development in our laboratory. The setup includes the construction of a rigid coil–sensor assembly for calibration and a phantom-based OPM-MEG measurement system to evaluate co-registration accuracy under realistic electromagnetic conditions. These efforts will enable quantitative comparison between simulated and real measurements, providing a more complete understanding of the algorithm’s stability and translational feasibility.

In future work, we plan to significantly expand the parameter space by incorporating a wider variety of coil geometries, sensor layouts, and noise levels. Moreover, we aim to validate the proposed method using experimental OPM-MEG data obtained from human participants to further demonstrate its practical applicability and generalizability. While the current findings are primarily simulation-based, their practical effectiveness must be confirmed through physical experiments. To this end, future studies will focus on constructing an actual RCS system, acquiring real OPM measurements, and evaluating performance under realistic electromagnetic conditions. In addition, further extensions will explore multimodal fusion approaches—such as integrating optical tracking data with electromagnetic modeling—to establish a more robust and adaptive co-registration framework.

## 5. Conclusions

This work addresses the vulnerability of OPM-MEG sensor co-registration to geometric uncertainties and assembly errors by proposing a method that combines magnetic dipole equivalent modeling with a rigid coil structure (RCS). The study systematically analyzes the primary sources of dipole approximation error (coil–sensor distance, angular coupling, and projection effects) and develops improved strategies that integrate geometric constraints with optimized objective functions.

In practical implementation, the assembly perturbations introduced in the simulations (±0.5 mm positional and ±0.5° angular deviations) were selected according to the manufacturing precision achievable with resin-based 3D-printed rigid helmets. Commercial stereolithography (SLA) printers and standard alignment fixtures typically provide dimensional accuracy better than 0.5 mm and angular repeatability within 0.5°. Therefore, the simulated perturbation range realistically represents the expected mechanical tolerances of the RCS assembly. The simulation results showing sub-millimeter and sub-degree localization errors indicate that the proposed RCS-based method remains robust within these practical fabrication limits, ensuring that its performance is consistent with the physical precision achievable in real OPM-MEG hardware.

Simulation results show that under ideal conditions, the proposed method achieves position errors below 1 mm and orientation errors below 1°. When assembly perturbations of up to 0.5 mm in position and 0.5° in orientation are introduced, both the weighted Frobenius norm and SSIM still maintain position errors within 0.6 mm and orientation errors within 0.6°, while effectively suppressing large outliers. These results significantly outperform the standard Frobenius norm, confirming the effectiveness of the proposed framework in improving accuracy and robustness.

Despite the current simulation-based validation, this study establishes a rigorous theoretical foundation for accurate and robust OPM-MEG sensor co-registration using rigid coil structures. The proposed framework demonstrates high feasibility and robustness through systematic simulations and provides a promising pathway for engineering implementation. Future research will focus on constructing a physical RCS–OPM system, performing coil–sensor calibration experiments, and validating the method using both phantom-based and human OPM data. These efforts will bridge the gap between simulation and experiment, thereby enhancing the practical applicability and translational value of the proposed approach.

Building upon these findings, this work provides a solid theoretical and methodological basis for achieving high-precision alignment in OPM-MEG systems using magnetic dipole modeling. The framework elucidates the error mechanisms inherent in the magnetic dipole approximation and introduces a co-registration method that combines high accuracy with strong robustness. Future research will extend the computational analysis to more diverse parameter sets and real OPM experimental data and will further explore integration with optical tracking, multimodal co-registration, and large-scale array applications to enhance the system’s practicality and engineering relevance.

## Figures and Tables

**Table 1:** Simulation of scalp-attached equivalent magnetic dipoles (85 × 35 = 2975 groups).

|*E*|	Number of Point	Proportion
<0.01	2387	80.24%
<0.02 <0.03 <0.04 <0.05	2606 2690 2756 2799	87.60% 90.42% 92.64% 94.08%

**Table 2:** Simulation of RCS equivalent magnetic dipoles (85 × 35 = 2975 groups).

|*E*|	Number of Point	Proportion
<0.01	1554	52.24%
<0.02 <0.03 <0.04 <0.05	1629 1648 1655 1657	54.76% 55.39% 55.63% 55.70%

**Table 3:** Simulation results of sensor positioning and orientation (ideal conditions).

Objective Function	Average Positioning Error (mm)	Average Angle Error (°)
**Frobenius norm**	Group 1: 0.18 Group 2: 0.19 Group 3: 0.18	Group 1: 0.55 Group 2: 0.49 Group 3: 1.02
**Weighted**	Group 1: 0.13 Group 2: 0.16 Group 3: 0.16	Group 1: 0.27 Group 2: 0.15 Group 3: 0.58
**SSIM**	Group 1: 0.16 Group 2: 0.18 Group 3: 0.17	Group 1: 0.36 Group 2: 0.34 Group 3: 0.49

**Table 4:** Simulation results of sensor positioning and orientation with assembly perturbations.

Objective Function	Average Positioning Error (mm)	Average Angle Error (°)
**Frobenius norm**	Group 1: 0.60 Group 2: 0.52 Group 3: 0.93	Group 1: 0.55 Group 2: 0.49 Group 3: 1.02
**Weighted**	Group 1: 0.26 Group 2: 0.24 Group 3: 0.56	Group 1: 0.27 Group 2: 0.15 Group 3: 0.58
**SSIM**	Group 1: 0.35 Group 2: 0.27 Group 3: 0.47	Group 1: 0.36 Group 2: 0.34 Group 3: 0.49

## Data Availability

This study employs simulated data. As such, no new empirical data were created or collected. The simulation datasets supporting the conclusions are not publicly archived but can be made available by the corresponding author upon justified request.

## Appendix A

**Table A1:** Summary of Symbols and Parameters.

Symbol	Description	Unit
$\mu_{0}$	permeability of vacuum	4π × 10^−7^ H/m
$r_{s}$	position vector of the sensor	mm
$r_{c}$	position vector of coil	mm
$n_{s}$	unit vector of the sensor’s sensitive axis	_
$m_{c}$	Magnetic moment vector of the coil	A·m^2^
$r_{c,s}$	position vector of the sensor	mm
$\theta_{m}^{c,s}$	angle between the coil’s magnetic moment direction and the observation direction	rad
$\theta_{n}^{c,s}$	angle between the sensor’s sensitive axis and the observation direction	rad
$\theta_{c}^{s}$	angle between the magnetic moment and the sensor’s sensitive axis	rad
$\theta_{s}$	polar angle of the sensor’s spherical coordinates	rad
$\phi_{s}$	azimuth angle of the sensor’s spherical coordinates	rad
$x_{s}$	*X*-axis coordinate of the sensor in the Cartesian coordinate system	mm
$y_{s}$	*Y*-axis coordinate of the sensor in the Cartesian coordinate system	mm
$z_{s}$	*Z*-axis coordinate of the sensor in the Cartesian coordinate system	mm
I(t)	instantaneous current of the coil	A
k	scaling factor proportional to the coil driving current	_
$B_{c,s}$	measured magnetic field	T
$B_{c,s}^{*}$	predicted or reference magnetic field	T
E	quantify the fit between the dipole-model field and the ideal magnetic field	_

## Appendix B

SSIM Parameter Settings

The Structural Similarity Index Measure (SSIM) used in this study follows the standard formulation proposed by Wang et al. [30]. To ensure reproducibility, the parameter settings adopted in the simulations are explicitly listed here. The SSIM between the simulated and reference magnetic field matrices was computed using the default weighting parameters α=β=γ=1 and the constants defined as $C_{1}={\left(0.01L\right)}^{2}$, $C_{2}={\left(0.03L\right)}^{2}$, $C_{3}=\frac{C_{2}}{2}$, where L denotes the dynamic range of the magnetic field data. These values are consistent with the standard configuration recommended by Wang et al. and ensure comparability with other SSIM-based evaluations in related studies.
